# WT1‐interacting protein inhibits cell proliferation and tumorigenicity in non‐small‐cell lung cancer via the AKT/FOXO1 axis

**DOI:** 10.1002/1878-0261.12462

**Published:** 2019-02-22

**Authors:** Zhiqiang Wu, Minghan Qiu, Zeyun Mi, Maobin Meng, Yu Guo, Xiangli Jiang, Jiaping Fang, Hui Wang, Jinlin Zhao, Zhuang Liu, Dong Qian, Zhiyong Yuan

**Affiliations:** ^1^ Department of Radiation Oncology Key Laboratory of Cancer Prevention and Therapy National Clinical Research Center for Cancer Tianjin's Clinical Research Center for Cancer Tianjin Medical University Cancer Institute & Hospital China; ^2^ Department of Oncology Tianjin Union Medical Center China; ^3^ Department of Biochemistry and Molecular Biology College of Basic Medical Science Tianjin Medical University China; ^4^ Department of General Surgery The First Affiliated Hospital Sun Yat‐sen University Guangzhou China; ^5^ Department of Thoracic Medical Oncology Tianjin Medical University Cancer Institute & Hospital China; ^6^ Community Health Service Center Tianjin China

**Keywords:** AKT, cell proliferation, FOXO1, non‐small cell lung cancer, NSCLC, WTIP

## Abstract

Lung cancer is the most common cancer and the leading cause of cancer‐related death worldwide; hence, it is imperative that the mechanisms underlying the malignant properties of lung cancer be uncovered in order to efficiently treat this disease. Increasing evidence has shown that WT1‐interacting protein (WTIP) plays important roles both physiologically and pathologically in humans; however, the role of WTIP in cancer is unknown. Here, we investigated the role and mechanism of WTIP in cell proliferation and tumorigenesis of non‐small‐cell lung cancer (NSCLC). We report that WTIP is a tumor suppressor in human NSCLC. We found that WTIP expression was significantly reduced in both NSCLC cell lines and clinical specimens compared to that in normal controls; this reduction was largely attributed to promoter hypermethylation. Downregulation of WTIP significantly correlates with poor prognosis and predicts a shorter overall survival and progression‐free survival among NSCLC patients. Moreover, ectopic overexpression of WTIP dramatically inhibits cell proliferation and tumorigenesis *in vitro* and *in vivo*; conversely, depletion of WTIP expression shows the opposite effects. Mechanistically, WTIP impairs AKT phosphorylation and activation, leading to enhanced expression and transcriptional activity of FOXO1, which further increases p21Cip1 and p27Kip1, and decreases cyclin D1, which consequently results in cell cycle arrest. Collectively, the results of the current study indicate that WTIP is an important proliferation‐related gene and that WTIP expression may represent a novel prognostic biomarker for NSCLC.

**Abbreviations**
BrdU: bromodeoxyuridine
CDK: cyclin‐dependent kinase
FOXO1: forkhead box O1
GAPDH: glyceraldehyde‐3‐phosphate dehydrogenase
GSEA: gene set enrichment analysis
GSK‐3β: glycogen synthase kinase 3 beta
LUAD: lung adenocarcinoma
LUSC: lung squamous cell carcinoma
MTT: 3‐(4,5‐dimethylthiazol‐2‐yl)‐2,5‐dimethyltetrazolium bromide
NSCLC: non‐small‐cell lung cancer
OS: overall survival
PFS: progression‐free survival
PPS: postprogression survival
siRNA: small interfering RNA
WTIP: WT1‐interacting protein

## 1 Introduction

Normal tissues maintain their population and normal architecture and function by strictly controlling the production and release of growth‐promoting signals that guide entry into and progression through the cell cycle. However, these signals are deregulated in cancer cells. Carcinogenesis is a complex multistep process characterized by the progressive accumulation of genetic and epigenetic alterations, which ultimately lead to uncontrolled cell proliferation and tumor formation. The ability to sustain chronic proliferation is the most fundamental trait of cancer cells (Hanahan and Weinberg, 2011). Thus, it is important to elucidate the underlying mechanisms of sustained cell proliferation in cancer cells to restrict tumor growth and provide cancer treatment.

AKT mediates multiple critical signal transduction processes, including cell proliferation and survival, by phosphorylating downstream factors. FOXO1 was found to be one of the most important substrates of AKT. Upon activation by insulin or growth factors, AKT is phosphorylated and activated, which subsequently phosphorylates FOXO1 protein. Phosphorylated FOXO1 is then polyubiquitinated and degraded through the proteasome pathway (Aoki *et al*., 2004; Tang *et al*., 1999). FOXO1, a transcriptional factor, can directly target the promoter of p27Kip1 and activate its expression (Medema *et al*., 2000). FOXO1 can also transactivate the expression p21Cip1 by forming complexes with SMAD3 and SMAD4 (Seoane *et al*., 2004). Moreover, cyclin D1 was demonstrated to be inhibited by FOXO proteins (Schmidt *et al*., 2002). Thus, AKT/FOXO1 signaling plays a pivotal role in cell cycle progression, which has been well studied (Li *et al*., 2009; Xia *et al*., 2012).

WT1‐interacting protein (WTIP) was originally identified as a coregulator of WT1 (Srichai *et al*., 2004). Wtip regulates basal body organization and cilia growth in *Drosophila* (Chu *et al*., 2016) and is required for proepicardial organ specification and cardiac left/right asymmetry in zebrafish (Powell *et al*., 2016) and neural crest development in *Xenopus* (Langer *et al*., 2008). Several studies have reported that WTIP has important roles in renal development, the maintenance of glomerular podocytes, and the pathology of chronic kidney diseases. After podocyte injury, WTIP shuttles from the slit diaphragm into the nucleus and suppresses WT1 activity, whose function is required for a normal podocyte phenotype (Rico *et al*., 2005; Srichai *et al*., 2004). WTIP is required for the stable assembly of podocyte adherens junctions and cell–matrix contacts. Podocytes with WTIP knocked down failed to form stable adherens junctions and exhibited disordered F‐actin structures (Kim *et al*., 2012). In addition, WTIP gene haploinsufficiency was reported to be responsible for hypospadias in humans (Gana *et al*., 2012). These studies indicate that WTIP plays important roles in both physiological and pathological conditions. However, there are little data regarding whether WTIP has an important role in cancer development and progression, which needs to be elucidated.

Here, we found that WTIP is dramatically downregulated in non‐small‐cell lung cancer (NSCLC) and that this downregulation is associated with poor prognosis in patients. We also demonstrated that WTIP could inhibit cell proliferation and tumorigenesis *in vitro* and *in vivo*. Mechanistically, WTIP suppresses AKT activation and enhances the transcriptional activity of FOXO1, which consequently increases the expression of cyclin‐dependent kinase inhibitors p21Cip1 and p27Kip1, decreases the expression of cyclin D1, and induces cell cycle arrest. Collectively, these findings suggest the important roles of WTIP in NSCLC and indicate that WTIP may be a potential target for this disease.

## 2 Materials and methods

### 2.1 Patient information and tissue specimens

Patient consent was informed and written. Patient consent and approval from the Institutional Research Ethics Committee of Tianjin Cancer Institute and Hospital were obtained for the use of the clinical materials for research purposes.

A total of 94 archived paraffin‐embedded NSCLC samples, which were histopathologically and clinically diagnosed at the Tianjin Medical University Cancer Institute and Hospital from 2011 to 2013, were used in the current study. Ten primary NSCLC tumor and adjacent nontumor tissue sample pairs were obtained from the Department of Pathology, Tianjin Medical University Cancer Hospital. Fresh tumor tissues were snap‐frozen in liquid nitrogen in the operating room and then stored at −80 °C. The tumor tissues were histopathologically and clinically diagnosed using both fresh and formalin‐fixed, paraffin‐embedded tumor tissues. All fresh samples were confirmed by hematoxylin and eosin (H&E) staining in frozen sections for the histopathological analysis, and paired tumor and adjacent nontumor tissues were dissected and divided into two parts for RNA and protein studies. Clinical information relating to the samples is summarized in Table 1. The study methodologies conformed to the standards set by the Declaration of Helsinki.

**Table 1:** Clinicopathological characteristics of patient samples and WTIP expression levels in NSCLC and the correlation between WTIP expression and the clinicopathological characteristics of NSCLC patients

Patient characteristics	WTIP expression		Chi‐square test *P*‐value	Fisher's exact test *P*‐value
	Low	High		
Gender				
Male	39	34	0.491	0.620
Female	13	8		
Age (year)				
≥ 60	30	24	0.957	1.000
< 60	22	18		
Clinical stage				
I	11	17	0.035	0.029
II	10	12		
III	29	13		
IV	2	0		
T classification				
T_1_	10	16	0.051	0.042
T_2_	25	21		
T_3_	15	5		
T_4_	2	0		
N classification				
N_0_	20	24	0.047	0.031
N_1_	4	7		
N_2_	27	11		
N_3_	1	0		
M classification				
Yes	2	0	0.199	0.500
No	50	42		
Histological differentiation				
Well	7	7	0.909	0.919
Moderate	29	21		
Poor	17	13		

### 2.2 Immunohistochemistry

Immunohistochemistry was performed as described previously (Xia *et al*., 2012). Anti‐WTIP (SAB1303504; Sigma‐Aldrich, St. Louis, MO, USA) and anti‐Ki‐67 (8480; Cell Signaling Technology, Danvers, MA,USA) antibodies were used. The sections were reviewed independently by two blinded observers and scored based on both the proportion of positively stained tumor cells and the intensity of the staining. The proportion of positively stained tumor cells was scored as follows: 0, no positive tumor cells; 1, < 10%; 2, 10–50%; 3, 51–75%; and 4, > 75%. The intensity of staining was recorded according to the following criteria: 0, no staining; 1, weak staining (light yellow); 2, moderate staining (yellowish brown); and 3, strong staining (brown). The staining index was calculated as the proportion of positive cells × staining intensity score.

### 2.3 Plasmids and siRNA

The human WTIP ORF was PCR‐amplified from the cDNA of BEAS‐2B cell mRNA and cloned into SpeI/EcoRI sites of the pLVX‐IRES‐neo lentiviral vector containing an N‐terminal tagged FLAG epitope; the vector was validated by sequencing. pLKO.1‐puro‐shWTIP (TRCN0000239373) was originally from MISSION™ shRNA Libraries (Sigma‐Aldrich) and kindly provided by S. Cen (Institute of Medicinal Biotechnology, Chinese Academy of Medical Sciences and Peking Union Medical College) as a gift. The reporter plasmid for quantitatively detecting the transcriptional activity of FOXO was generated as previously described (Lin *et al*., 2011; Tang *et al*., 1999). FOXO1 siRNA was purchased from RiboBio Co., Ltd (Guangzhou, China), and the target sequence is GCCCUGGCUCUCACAGCAA.

### 2.4 Cell culture and establishment of stable cell lines

Non‐small‐cell lung cancer cell lines, including A549, H460, H520, H1299, H292, and PC‐9, were cultured in RPMI 1640 medium (Gibco, Grand Island, NY, USA). 293FT cells were cultured in DMEM (Gibco). The immortalized lung epithelial cell line BEAS‐2B was cultured in BEBM medium (LONZA, Basel, Switzerland).

For establishment of stable cell lines, plasmids (pLVX‐IRES‐neo‐Flag‐WTIP, pLKO.1‐puro‐shWTIP, and the control vectors) were transfected in 293FT to produce lentivirus. A549 and H460 cells were infected with the indicated lentivirus for 48 h and then selected with medium containing neomycin (500 μg·mL^−1^) or puromycin (1 μg·mL^−1^). All stable cell lines were selected with the indicated antibiotics at a high concentration for over 1 week and maintained in medium containing a low concentration of the indicated antibiotics.

### 2.5 Western blotting and antibodies

Western blotting was conducted as previously reported (Wu *et al*., 2018). Antibodies against AKT (4691), phospho‐AKT (2965), GSK‐3β (12456), phospho‐GSK‐3β (9323), FOXO1 (2880), phospho‐FOXO1 (9461), p27 (3686), p21 (2947), Rb (9309), and phospho‐Rb (8516) were purchased from Cell Signaling Technology. Anti‐cyclin D1 (TA801655) was purchased from ORIGENE (Rockville, MD, USA). WTIP (SAB1411722) and α‐tubulin (T9026) antibodies were obtained from Sigma‐Aldrich. Glyceraldehyde‐3‐phosphate dehydrogenase (GAPDH) (sc‐365062) antibody was purchased from Santa Cruz Biotechnology (Santa Cruz, CA, USA).

### 2.6 RNA extraction and reverse transcription and real‐time PCR

Total RNA was extracted using TRIzol reagent (Invitrogen, Carlsbad, CA, USA), and cDNA was synthesized using M‐MLV Reverse Transcriptase (Promega, Madison, WI,USA). Amplification and real‐time analysis were performed with a Bio‐Rad CFX96 system (Bio‐Rad, Hercules, CA, USA) using FastStart Universal SYBR Green Master (Roche, Basel, Switzerland) following the manufacturer's instructions. Transcript levels were normalized to those of the housekeeping gene GAPDH. The relative mRNA levels were calculated according to the comparative *C*_t_ (ΔΔ*C*_t_) method, where *C*_t_ represents the threshold cycle for each transcript. The specific primers used for quantitative PCR assays were as follows: 5′‐GCGGGACTACTTCGGCATT‐3′, 5′‐CTCTCCCACACGAGTCGCA‐3′ for WTIP; 5′‐AACTACCTGGACCGCTTCCT‐3′, 5′‐CCACTTGAGCTTGTTCACCA‐3′ for cyclin D1; 5′‐CGATGCCAACCTCCTCAACGA‐3′, 5′‐TCGCAGACCTCCAGCATCCA‐3′ for P21; 5′‐TGCAACCGACGATTCTTCTACTCAA‐3′, 5′‐CAAGCAGTGATGTATCTGATAAACAAGGA‐3′ for P27; and 5′‐ACCACAGTCCATGCCATCAC‐3′, 5′‐TCCACCACCCTGTTGCTGTA‐3′ for GAPDH.

### 2.7 Bisulfite genomic sequencing

Genomic DNA from different cells was bisulfite‐modified and purified with an EpiTect Bisulfite Kit (QIAGEN, Duesseldorf, Germany) according to the manufacturer's instructions. Bisulfite‐treated DNA was amplified with bisulfite‐sequencing PCR (BSP) primers targeted to the WTIP promoter. The primer sequences were TTAGTTGTTTTTAGTTTAGTTTG (forward) and AAAAAATATAAACTCCCAAA (reverse). PCR products were purified and cloned into the pMD19‐T Vector System (TaKaRa, Dalian, China). Three single colonies of each sample were selected for plasmid extraction and sequenced to determine the DNA methylation status.

### 2.8 MTT assay

Cells were seeded in 96‐well plates at an initial density of 1000 cells/well. At each time point, 20 μL of filter‐sterilized MTT reagent was added to the cell culture. After 4 h of incubation at 37 °C, the supernatant was discarded, and 150 μL of dimethyl sulfoxide (DMSO) was added (Sigma‐Aldrich). The absorbance was measured at 570 nm, with 655 nm as the reference wavelength. All experiments were performed in three biological duplicates at least three times.

### 2.9 Flow cytometry analysis

Flow cytometry was performed as described previously (Lin *et al*., 2011). Briefly, asynchronously growing cells were fixed with 70% ethanol in PBS, stained with PI (50 μg·mL^−1^), and analyzed by flow cytometry (FACSCalibur; BD Biosciences, San Jose, CA, USA). At least 10 000 cells per sample were analyzed. The cell cycle distribution was assessed using modfit (Verity Software House, Topsham, ME, USA).

### 2.10 BrdU incorporation and immunofluorescence

Cells (4 × 10^4^) were plated on coverslips and incubated overnight. The cells were then incubated with bromodeoxyuridine (BrdU) for 1 h and stained with anti‐BrdU antibody (sc‐32323; Santa Cruz) according to the manufacturer's instructions. Gray‐level images were acquired under a laser scanning microscope (axio imager.z2; Carl Zeiss Co. Ltd, Jena, Germany). Ten randomly selected fields at 200× magnification were used to quantify the percentage of BrdU‐positive cells.

### 2.11 Luciferase reporter assay

Cells (8 × 10^4^) were seeded in each well of a 24‐well plate and cultured overnight. The cells were then cotransfected with 1 ng pRL‐TK Renilla plasmid (as a reference) and 500 ng of either pLVX‐FLAG‐WTIP or pLKO.1‐shWTIP plus 100 ng p3x IRS‐Luc plasmid using Lipofectamine 2000 (Invitrogen). The media were replaced at 6 h after transfection, and luciferase and Renilla signals were measured 24 h (for 293FT cells) or 48 h (for A549 cells) after transfection using a Dual Luciferase Reporter Assay Kit (Promega).

### 2.12 Anchorage‐independent growth assay

Base layers of basic RPMI 1640 medium (2 mL) containing 0.6% Difco Noble Agar (BD Biosciences) were set in each well of a 6‐well cell culture plate. This was further overlaid with 2 mL of 0.3% agar containing a suspension of 3 × 10^4^ single cells in RPMI 1640 plus 20% FBS. The colony images were photographed under a microscope and quantified on day 10 for H460 cells and day 15 for A549 cells. Colonies larger than 0.1 mm in diameter were scored. Because not all colonies were spherical, the longest diameter of each colony was measured. All cultures were performed in triplicate, and the average number of at least 10 randomly selected microscopic fields was presented.

### 2.13 Xenografted tumor model

Female BALB/c‐nu mice (4–5 weeks old) were purchased from the Chinese Academy of Medical Sciences and were housed in specific pathogen‐free facilities on a 12‐h light/dark cycle. All experimental procedures were approved by the Institutional Animal Care and Use Committee of Tianjin Medical University. For tumor cell implantation, 6 × 10^6^ cells suspended in 0.1 mL of PBS were subcutaneously injected into nude mice. After the development of palpable tumors, the tumor volume (*V*) was monitored every 3 days and calculated with the formula *V* = 0.5 × Length × Width^2^. On day 42, animals were euthanized, and the tumors were excised and weighed.

### 2.14 Statistical analysis

Statistical analyses were carried out using the spss 19.0 (IBM, Armonk, NY, USA) statistical software package or graphpad prism 5 software (GraphPad Software, San Diego, CA, USA). The relationship between WTIP expression and the clinicopathological characteristics was analyzed by the chi‐square test. Survival curves were plotted by the Kaplan–Meier method and compared using the log‐rank test. Survival data were evaluated using univariate and multivariate Cox regression analyses. Two‐tailed, unpaired Student's *t*‐test was used for comparisons between groups for statistical significance. A *P*‐value < 0.05 in all cases was considered statistically significant.

## 3 Results

### 3.1 WTIP is downregulated in NSCLC resulting from promoter hypermethylation

**Figure 1:**
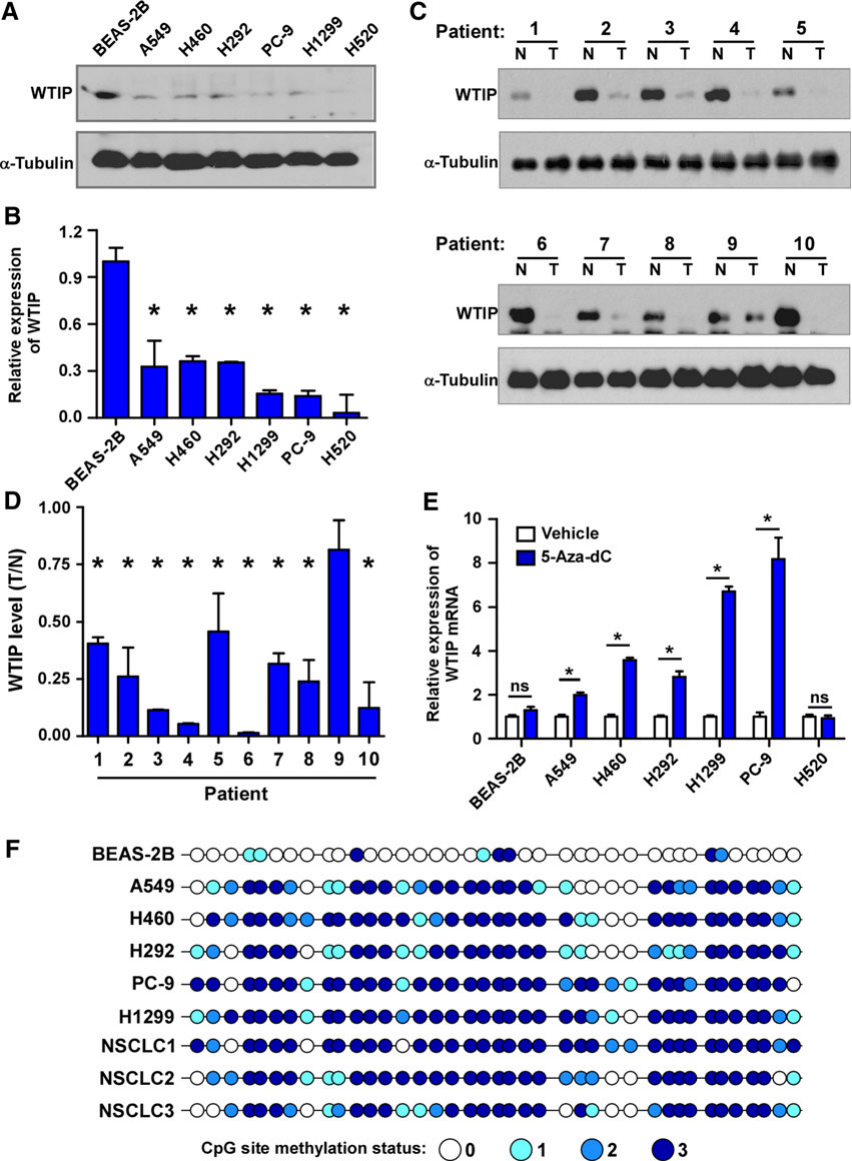
Promoter methylation leads to WTIP downregulation in NSCLC. Western blotting (A, C) and real‐time PCR (B, D) analysis of WTIP expression in NSCLC cell lines and paired NSCLC tumor and nontumor tissues. (E) Real‐time PCR analysis of WTIP mRNA levels in the indicated cells treated with vehicle or 5‐Aza‐dC for 72 h. Error bars represent the mean ± SD obtained from three independent experiments. *P < 0.05, unpaired *t*‐test. (F) Bisulfite genomic sequencing of three individual clones reveals the methylation status of CpG islands in the WTIP promoter in the indicated specimens. The methylation status of the CpG sites from three individual clones was distinguished by different shades of blue as indicated. N, nontumor. T, tumor.

First, WTIP expression was detected. Compared to the lung epithelial cell line BEAS‐2B, NSCLC cell lines showed significantly downregulated WTIP expression at both the protein and mRNA levels (Fig. 1A,B). Comparative analysis showed that the mRNA and protein levels of WTIP were also differentially repressed in almost all NSCLC tumor tissues compared with those in matched adjacent nontumor tissues (Fig. 1C,D). Furthermore, analysis with the publicly available website, GEPIA (http://gepia.cancer-pku.cn/), showed that WTIP was significantly downregulated in both lung adenocarcinoma (LUAD) and lung squamous cell carcinoma (LUSC) compared to the observed levels in the normal control (Fig. S1A). Data from The Cancer Genome Atlas (TCGA) database also showed that WTIP expression is significantly downregulated in tumor tissues compared to matched normal tissue from patients with LUAD or LUSC (Fig. S1B). These results collectively demonstrated that WTIP is downregulated in NSCLC.

Repressing gene expression by methylation of the promoter region has been well demonstrated and reviewed (Klose and Bird, 2006). A CpG island‐rich region of the human WTIP promoter was identified in the UCSC Genome Browser (Fig. S1C). To investigate whether WTIP downregulation results from the DNA methylation of this promoter region, we treated the cells with the DNA methyltransferase inhibitor 5‐Aza‐dC for 72 h and found that inhibiting DNA methylation significantly promoted WTIP expression, especially in cells with endogenously low levels of WTIP, such as H1299 and PC‐9 cells. However, in BEAS‐2B cells, WTIP expression was not affected (Fig. 1C). Interestingly, there was no increase in WTIP in H520 cells, which did not present detectable WTIP expression, after 5‐Aza‐dC treatment (Fig. 1A,B). Furthermore, the BSP assay results showed that the analyzed CpG islands were hypermethylated and that the levels of hypermethylation were inversely correlated with the level of WTIP in NSCLC cell lines and tumor specimens from NSCLC patients. In contrast, the majority of CpG islands in BEAS‐2B cells were not methylated (Fig. 1D). Taken together, these findings revealed that promoter methylation contributes to the downregulation of WTIP in NSCLC.

### 3.2 WTIP is an independent prognostic factor in NSCLC, and its downregulation is associated with poor prognosis in patients

**Figure 2:**
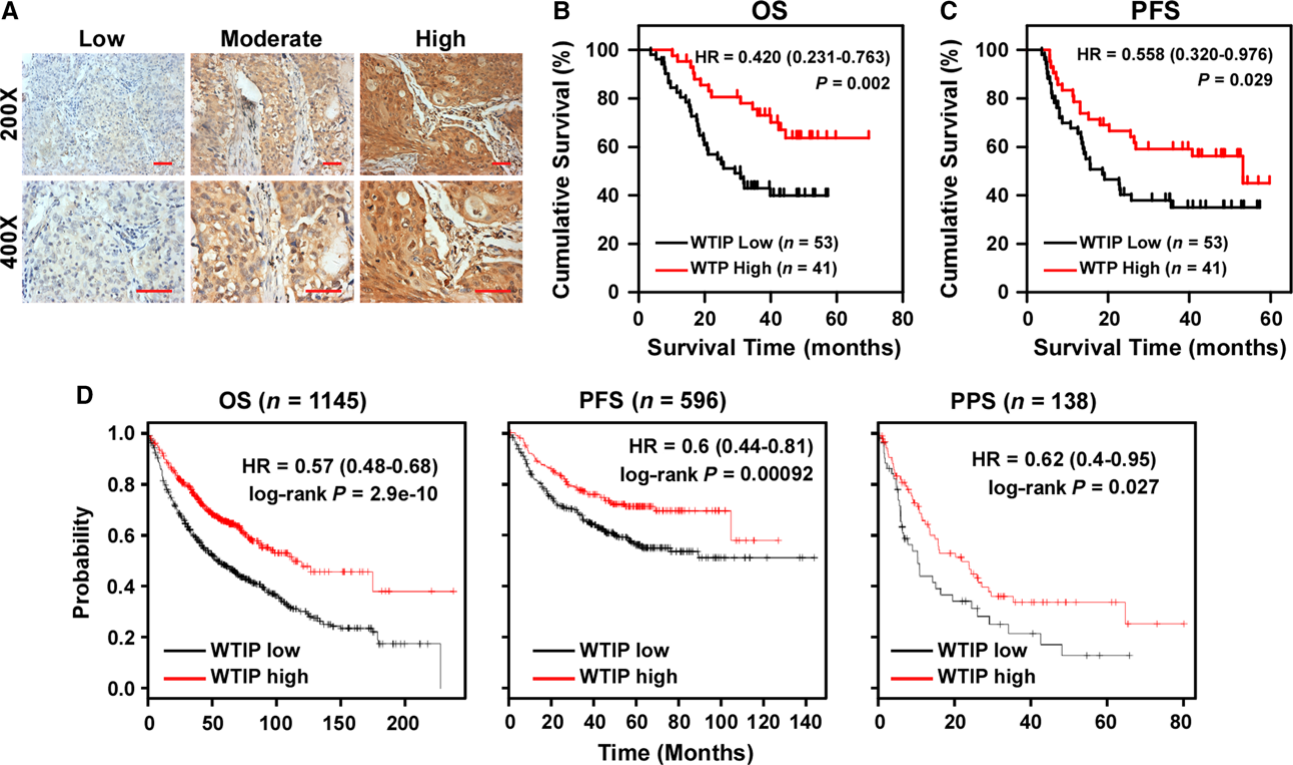
Downregulation of WTIP correlates with poor prognosis of NSCLC patients. (A) Representative cases of patient specimens with different levels of WTIP staining. Scale bars, 50 μm. (B, C) Kaplan–Meier analyses of OS (B) and PFS (C) of NSCLC patients stratified by WTIP expression levels. (D) Online Kaplan–Meier analyses of OS, PFS, and PPS of lung cancer patients with high or low levels of WTIP mRNA. Plots were generated using the publicly accessible tool KM Plotter. *P*‐value was calculated by log‐rank test.

To investigate the significance of downregulated WTIP in NSCLC, WTIP expression was examined by IHC in a cohort of NSCLC tissues. Representative pictures of NSCLC tissues with different WTIP expression levels are shown in Fig. 2A. Statistical analysis showed that WTIP expression correlated significantly with clinical stage (*P* = 0.035), T classification (*P* = 0.051), and N classification (*P* = 0.047) in NSCLC (Table 1). Kaplan–Meier survival analysis revealed that patients with low WTIP expression had worse overall survival (OS, HR = 0.420, *P* = 0.002) and progression‐free survival (PFS, HR = 0.558, *P* = 0.029) (Fig. 2B,C) than patients with high WTIP expression. Assessment from the publicly available KM Plotter database (http://kmplot.com/analysis/index.php?p=service&cancer=lung) showed that low WTIP expression correlated with worse OS, PFS, and postprogression survival (PPS) (Fig. 2D), which is consistent with and further validated our results. Moreover, univariate and multivariate analyses revealed that the clinical stage and WTIP expression were each recognized as an independent prognostic factor (Table 2). This suggests that WTIP expression may represent a novel prognostic biomarker for NSCLC.

**Table 2:** Univariate and multivariate analyses of various prognostic parameters in patients with NSCLC based on Cox regression models

Characteristics	Univariate analysis			Multivariate analysis		
	*P*	Relative risk	95% confidence interval	*P*	Relative risk	95% confidence interval
WTIP	0.006	0.407	0.215–0.771	**0.047**	0.510	0.263–0.990
T classification	0.087	1.393	0.953–2.037	0.593	1.134	0.716–1.796
N classification	0.006	1.540	1.131–2.096	0.720	1.138	0.561–2.309
M classification	0.002	10.377	2.280–47.235	0.108	4.099	0.734–22.898
Clinical stage	0.002	1.828	1.248–2.678	**0.014**	1.630	1.103–2.407

### 3.3 Overexpression of WTIP inhibits cell proliferation and induces G1/S phase arrest of NSCLC cells

**Figure 3:**
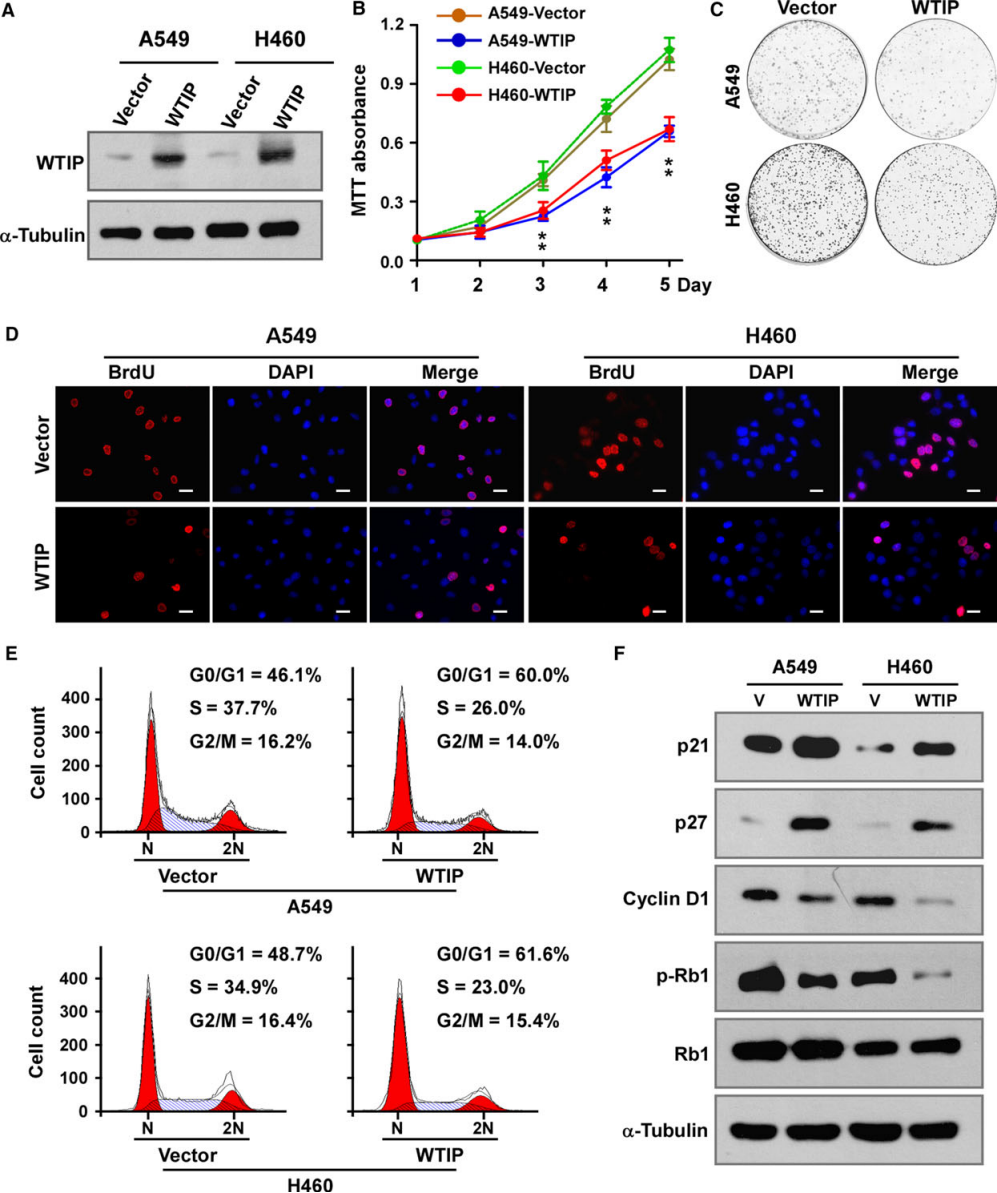
Overexpression of WTIP inhibits cell proliferation and induces cell cycle arrest in NSCLC cells. (A) Western blotting analysis of WTIP in the indicated cells with stable overexpression of WTIP. (B) MTT assay analysis of cell growth. Error bars represent the mean ± SD obtained from three independent experiments. *P < 0.05, unpaired *t*‐test. (C) Representative pictures of cell colonies originating from the indicated cells and stained with crystal violet. (D) Representative pictures of BrdU staining of the indicated cells. Pictures were taken at 400× magnification. Scale bars, 20 μm. (E) Cell cycle distribution as analyzed by flow cytometry. (F) Western blotting analyses of the expression of the cell cycle regulators p21, p27, cyclin D1, phosphorylated Rb (p‐Rb), and total Rb in vector control‐expressing and WTIP‐overexpressing cells. α‐Tubulin served as a loading control.

To determine the biological function of WTIP in NSCLC, WTIP was ectopically overexpressed in A549 and H460 cells (Fig. 3A and Fig. S2A). Interestingly, while culturing the cells, decreased cell proliferation and diminished growth of WTIP‐overexpressing cells were noticed; thus, these aspects were subsequently investigated. The MTT assay showed that WTIP overexpression significantly impaired cell proliferation in both cell lines (Fig. 3B), and these results were further confirmed by colony formation assay (Fig. 3C and Fig. S2B). Then, a BrdU incorporation assay was employed to investigate the mechanism by which this slowed growth occurs. As shown in Fig. 3D and Fig. S2C, WTIP overexpression dramatically decreased the fraction of BrdU‐positive A549 and H460 cells in the S‐phase fraction compared with their respective control cells (29.08% versus 46.15% and 23.80% versus 45.32%, respectively). Flow cytometry analysis revealed that compared to the vector control treatment, WTIP overexpression resulted in a significant increase in the percentage of cells in G0/G1 phase (60.0% versus 46.1% for A549 cells and 61.6% versus 48.7% for H460 cells) and a decrease in the percentage of cells in S phase (26.0% versus 37.7% for A549 cells and 23.0% versus 34.9% for H460 cells; Fig. 3E and Fig. S2D). Moreover, western blotting and real‐time PCR analyses revealed that WTIP overexpression significantly enhanced the expression of the cyclin‐dependent kinase (CDK) inhibitors p21Cip1 and p27Kip1, which are critical regulators of the G1–S transition (Ahmad *et al*., 2001; Harper *et al*., 1993; Toyoshima and Hunter, 1994), and impaired the expression of the CDK regulator cyclin D1 (Fig. 3F and Fig. S2E). As expected, the level of phosphorylated Rb, the downstream target protein of CDK, was shown to be suppressed in cells with WTIP overexpression, further supporting the notion that WTIP is involved in the regulation of NSCLC cell proliferation.

### 3.4 Depletion of WTIP releases cells from cell cycle arrest and promotes cell proliferation

**Figure 4:**
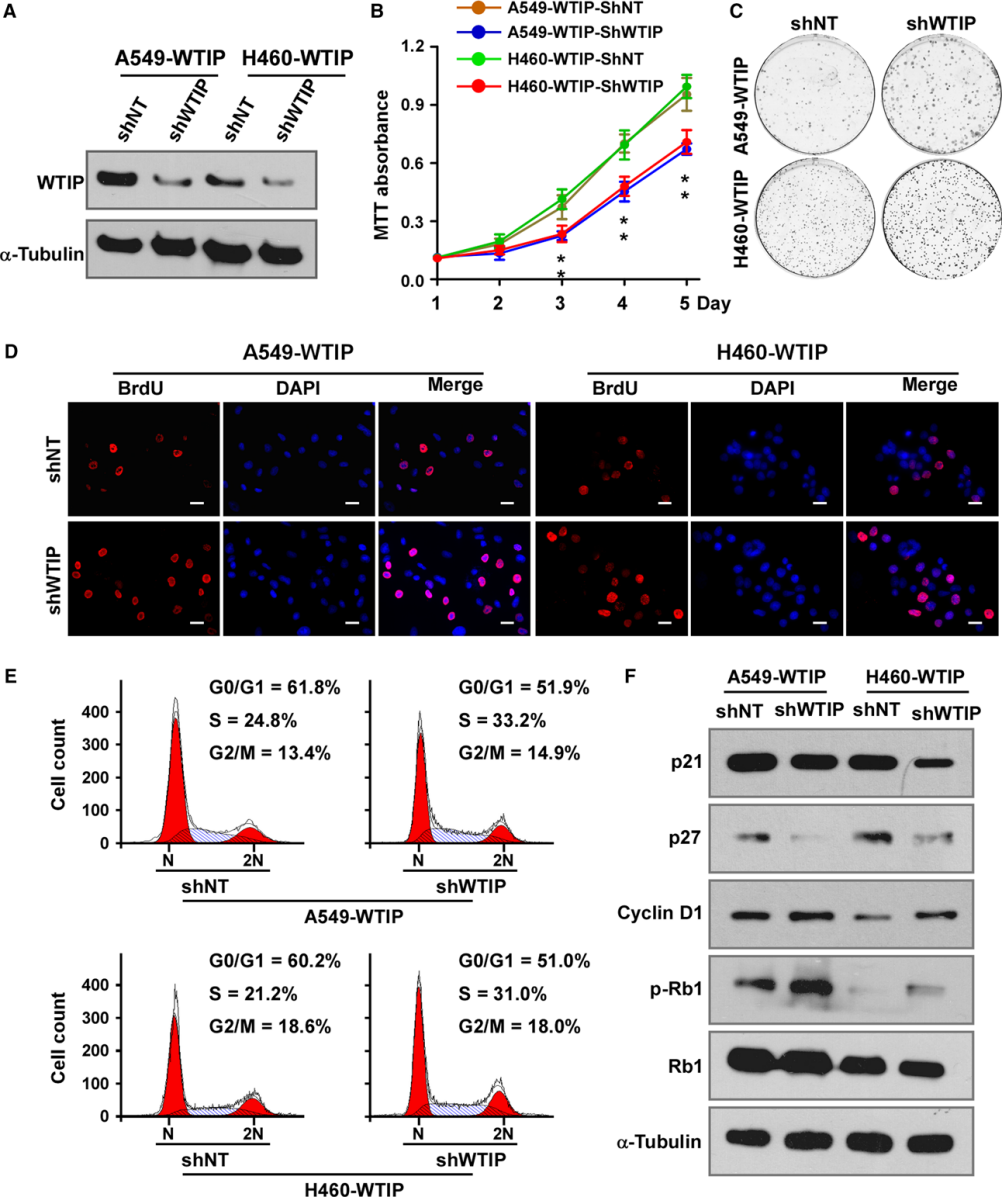
Depletion of WTIP promotes cell proliferation and cell cycle progression. (A) Western blotting analysis of WTIP in cell lines with stable expression of specific shRNAs. (B) MTT assay analysis of cell growth. Error bars represent the mean ± SD obtained from three independent experiments. *P < 0.05, unpaired *t*‐test. (C) Representative pictures of cell colonies originating from the indicated cells and stained with crystal violet. (D) Representative pictures of BrdU staining. Pictures were taken at 400× magnification. Scale bars, 20 μm. (E) Cell cycle distribution as analyzed by flow cytometry. (F) Western blotting analyses of the expression of p21, p27, cyclin D1, phosphorylated Rb (p‐Rb), and total Rb in the indicated cells. α‐Tubulin served as a loading control.

To further validate the role of WTIP in depressing proliferation, a specific shRNA targeting WTIP was employed to deplete WTIP in A549‐WTIP and H460‐WTIP cells (Fig. 4A and Fig. S3A). The MTT assay (Fig. 4B) and colony formation assay (Fig. 4C and Fig. S3B) showed that depleting WTIP restored the proliferation rate of A549‐WTIP and H460‐WTIP cells. The BrdU incorporation assay and cell cycle analysis by flow cytometry demonstrated that knockdown of WTIP released A549‐WTIP and H460‐WTIP cells from G1/S phase arrest, as indicated by the increase in the overall percentage of BrdU‐positive cells and percentage of cells in the S fraction and a decrease in the percentage of cells in the G0/G1 fraction (Fig. 4D,E, Fig. S3C,D). The expression of p21Cip1 and p27Kip1 was impaired, while the expression levels of cyclin D1 and phosphorylated Rb were enhanced by silencing WTIP expression (Fig. 4F and Fig. S3E). Overall, these results proved that WTIP inhibits cell proliferation and cell cycle progression in NSCLC cells.

### 3.5 WTIP reduces the tumorigenicity of NSCLC cells *in vitro* and *in vivo*

**Figure 5:**
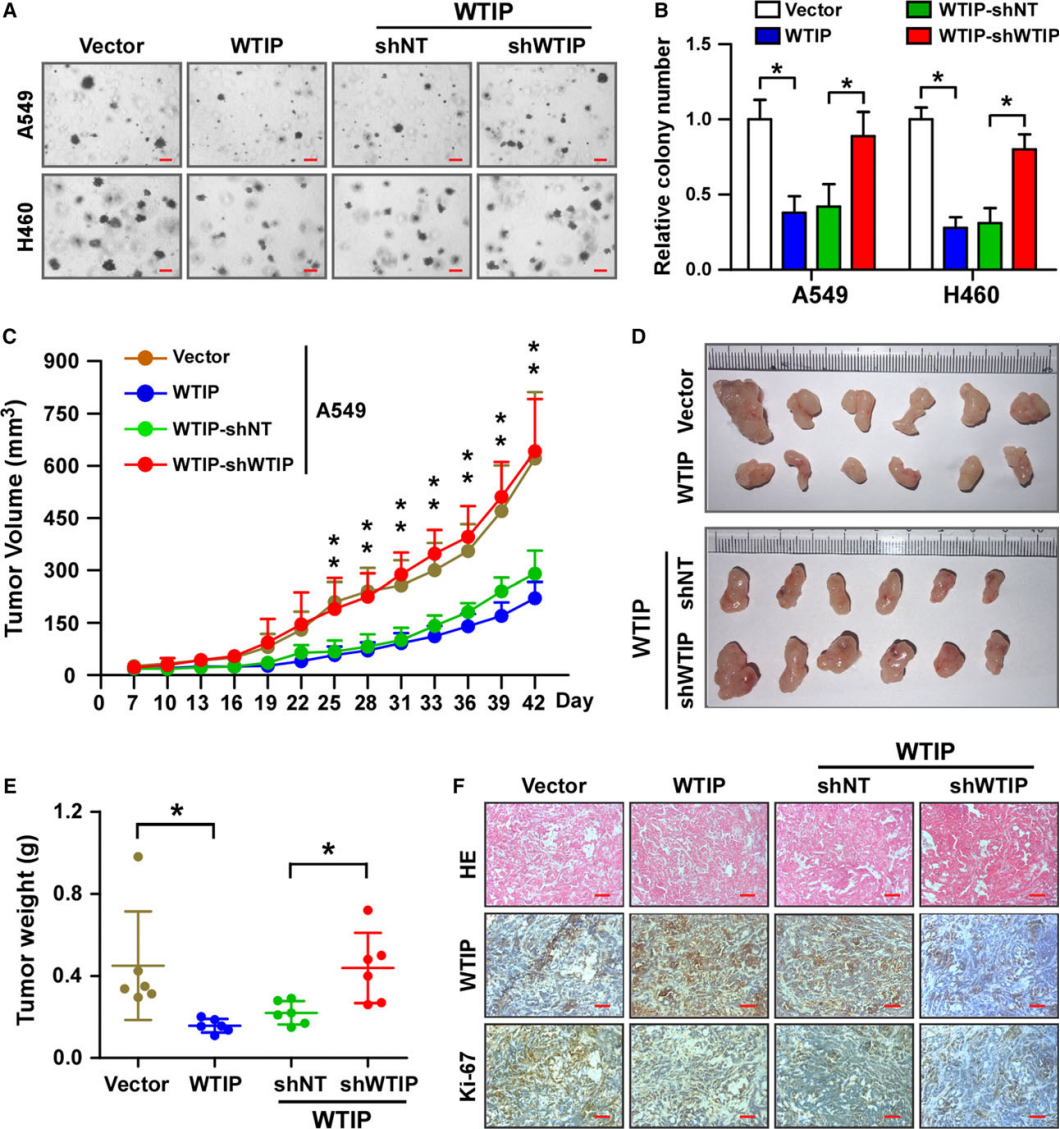
WTIP inhibits tumorigenicity of NSCLC *in vitro* and *in vivo*. Representative pictures (A) and quantification (B) of colony formation as analyzed by anchorage‐independent growth. Pictures were taken at 40× magnification. Scale bars, 0.2 mm. Error bars represent the mean ± SD obtained from three independent experiments. *P < 0.05, unpaired *t*‐test. (C–F) Xenograft model in nude mice. Different groups of A549 cells were subcutaneously injected into nude mice. (C) Growth curve of the tumor volumes measured on the indicated days. Error bars represent the mean ± SD. *P < 0.05, unpaired *t*‐test. (D and E) Representative pictures of tumor growth (D) and tumor weights (E) 42 days after inoculation. Error bars represent the mean ± SD. *P < 0.05, unpaired *t*‐test. (F) Representative pictures of H&E staining and IHC staining of WTIP and Ki‐67 in the indicated xenografted tumors. Scale bars, 50 μm.

Furthermore, the effect of WTIP on the tumorigenic activity of NSCLC cells was evaluated. Figure 5A,B shows that overexpression of WTIP significantly inhibited, while depleting WTIP derepressed the anchorage‐independent growth of both NSCLC cell lines as indicated by the reduction in the colony number on soft agar. Moreover, to determine whether WTIP could inhibit the tumorigenicity of NSCLC cells *in vivo*, A549 cells with WTIP overexpression or WTIP knockdown and the corresponding vector controls were subcutaneously implanted into nude mice (*n* = 6/group). The A549 cells with WTIP overexpression showed slower tumor growth than the cells containing the vector control (Fig. 5C). After 42 days, the sizes and weights of tumors from the A549‐WTIP cells were substantially lower than those from the control cells (Fig. 5D,E). Moreover, H&E and IHC staining showed that there were fewer Ki‐67‐positive cells in the tumors derived from A549‐WTIP cells than in the vector cells (Fig. 5F). However, cells with depleted WTIP, but not those with the nontarget control, exhibited a reversal in the effects of WTIP (Fig. 5C–F). Collectively, our results indicate that WTIP plays a pivotal role in inhibiting the tumorigenicity of NSCLC cells both *in vitro* and *in vivo*.

### 3.6 WTIP modulates cell proliferation and the cell cycle via AKT/FOXO1 signaling in NSCLC

**Figure 6:**
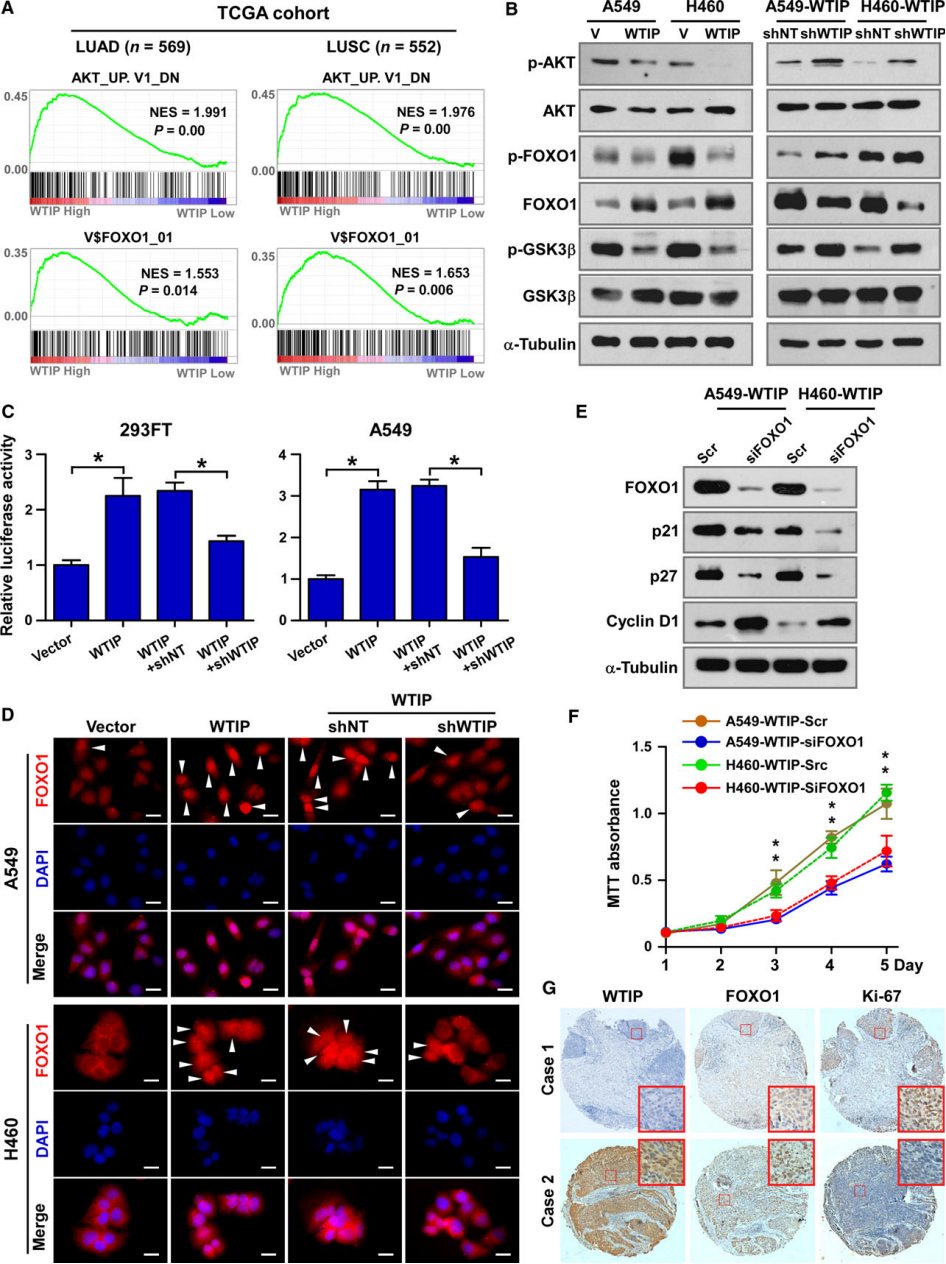
WTIP expression correlates with FOXO1 activation in NSCLC. (A) GSEA plot showing that WTIP expression is positively correlated with the AKT‐downregulated gene signature (AKT_UP. V1_DN) and FOXO1‐targeted gene signature (V$FOXO1_01) in the gene expression profiles of LUAD and LUSC patients from the TCGA database. (B) Western blotting analyses of the expression of phosphorylated AKT (p‐AKT), total AKT, phosphorylated FOXO1 (p‐FOXO1), total FOXO1, phosphorylated GSK‐3β (p‐ GSK‐3β), and total GSK‐3β. α‐Tubulin served as a loading control. (C) Relative FOXO1 luciferase reporter activity in 293FT and A549 cells transfected with the indicated plasmids. *P < 0.05, unpaired *t*‐test. (D) Representative pictures of FOXO1 immunostaining. White arrows, cell with nucleus retained FOXO1. Pictures were taken at 630× magnification. Scale bars, 20 μm. (E) Western blotting analyses of the expression of FOXO1, p21, p27, and cyclin D1. α‐Tubulin served as a loading control. (F) MTT assay analysis of cell growth of the indicated cells. Error bars represent the mean ± SD obtained from three independent experiments. *P < 0.05, unpaired *t*‐test. (G) IHC staining analysis of WTIP, FOXO1, and Ki‐67 expression in NSCLC patient specimens.

It has been well established that AKT/FOXO1 signaling regulates cell proliferation and the cell cycle by modulating the expression of p21Cip1, p27Kip1, and cyclin D1 (Dijkers *et al*., 2000; Nakamura *et al*., 2000; Schmidt *et al*., 2002; Seoane *et al*., 2004). To determine whether WTIP modulates the cell cycle and cell proliferation via AKT/FOXO1 signaling, gene set enrichment analysis (GSEA) was performed. The results showed that WTIP expression positively correlated with AKT inhibition (AKT_UP. V1_DN) and FOXO1‐activated (V$FOXO1_01) gene signatures in gene expression profiles of LUAD and LUSC patients obtained from the TCGA database (Fig. 6A).

AKT‐mediated phosphorylation of FOXO1 leads to the nuclear export and subsequent degradation of FOXO1 via the proteasome, which consequently inactivates the transcriptional activity of the protein (Aoki *et al*., 2004; Tang *et al*., 1999). Western blotting assays showed that ectopic expression of WTIP decreased the expression of phospho‐AKT (Thr308) and phospho‐FOXO1 (Ser256) and increased the total expression level of FOXO1. Knockdown of WTIP recovered the levels of phosphorylated AKT and FOXO1 proteins (Fig. 6B). In parallel, changes in the levels of phosphorylated GSK‐3β (a downstream target protein of AKT) were associated with the change in AKT phosphorylation, supporting the notion that WTIP contributes to the inhibition of AKT activation (Fig. 6B). Luciferase reporter activity analysis demonstrated that WTIP positively regulated FOXO1 transcriptional activity (Fig. 6C). Immunostaining and nuclear protein extraction assays showed that nuclear retention of FOXO1 protein was increased in cells that overexpressed WTIP and decreased in cells with depleted WTIP (Fig. 6D and Fig. S4A).

To further demonstrate that WTIP inhibits NSCLC proliferation via FOXO1, FOXO1 was knocked down by a specific siRNA in WTIP‐overexpressing cells. Knockdown of FOXO1 was shown to block the effects of WTIP on cell cycle and cell proliferation as indicated by the reduced expression of p21Cip1 and p27Kip1, the enhanced expression of cyclin D1 (Fig. 6E and Fig. S4B), and the recovery of the cell proliferation rate (Fig. 6F) in WTIP‐overexpressing cells. These data demonstrate that the transcriptional factor FOXO1 functions downstream of WTIP and mediates the effects of WTIP on the cell cycle and proliferation. Moreover, after analyzing a cohort of 94 NSCLC tissue specimens, WTIP levels were observed to be strongly positively correlated with FOXO1 expression and negatively correlated with Ki‐67 expression (Fig. 6G and Fig. S4C), which further supported the notion that WTIP inhibits cell proliferation and the cell cycle via FOXO1 in NSCLC clinical specimens.

## 4 Discussion

To date, the expression of WTIP in cancers has never been investigated. In this study, we reported for the first time that WTIP is significantly downregulated at both the mRNA and protein levels in NSCLC. Both genetic and epigenetic alterations of tumor suppressor and tumor‐related genes are involved in the pathogenesis of cancer. By analyzing the genomic DNA sequence of WTIP, a CpG island‐rich region of the human WTIP promoter was identified. Moreover, 5‐Aza‐dC treatment and the BSP assay demonstrated that the WTIP promoter region is hypermethylated in NSCLC (Fig. 1C,D), which accounts for the downregulation of WTIP in NSCLC. Interestingly, in the LUSC cell line H520, we could neither detect WTIP expression, if any (Fig. 1A and Fig. S1A), nor amplify the promoter region for sequencing (data not shown). WTIP is located at chromosome 19q13.11. By performing comparative genomic hybridization array analysis and real‐time PCR in 14 LUSC patients, Choi *et al*. (2007) reported that 19q13.11 is one of the most common deleted regions in this cancer. Thus, it is possible that WTIP is deleted in H520 cells. Accordingly, we speculate that both epigenetic and genetic alterations lead to WTIP downregulation in NSCLC. According to our results, promoter methylation might be the main cause of the reduced expression of WTIP in NSCLC cell lines; however, whether promoter methylation is observed in NSCLC clinical samples needs to be further confirmed.

WTIP was originally identified as a WT1‐interacting protein and repressed WT1‐dependent transcription in kidney (Srichai *et al*., 2004). Moreover, Xu *et al*. (2013) reported that WT1 promotes cell proliferation in NSCLC cell lines by upregulating cyclin D1 and p‐Rb *in vitro* and *in vivo*. Thus, we speculated that WTIP functions as a tumor suppressor and inhibits cell proliferation in NSCLC. Functional studies revealed that WTIP did inhibit the tumorigenicity of NSCLC *in vitro* and *in vivo* (Fig. 5). Interestingly, though our data showed that WTIP overexpression significantly inhibited cell proliferation, downregulated cyclin D1 and p‐Rb levels, and induced the expression of the CDK inhibitors p21Cip1 and p27Kip1 (Figs 3F and 4F), there are no inhibitory effects of WTIP on WT1 found in this study (Fig. S6). In contrast, we found that depressing AKT and further activating FOXO1 (Fig. 6) accounted for the cell proliferation‐ and tumorigenesis‐inhibiting roles of WTIP. This is unexpected and might be due to different models used.

WTIP together with LIMD1 and AJUBA constitutes the LIM protein subfamily of Ajuba proteins, which are characterized by a highly conserved carboxyl terminus with three highly related tandem LIM domains (the LIM region) and a variable proline‐rich amino‐terminal pre‐LIM region (Schimizzi and Longmore, 2015). Thus, members of this subfamily exhibit both shared and unique functions. For example, Ajuba proteins have been reported to participate in the regulation of Snail/Slug, microRNA‐mediated gene silencing, and Hippo signaling pathways with similar functions (Jagannathan *et al*., 2016; James *et al*., 2010; Langer *et al*., 2008). LIMD1 and AJUBA have been reported to regulate cell cycle and proliferation, however, with contrary roles. AJUBA interacts with Aurora‐A and promotes cell cycle progression. Depletion of AJUBA prevented Aurora‐A activation and inhibited mitotic entry (Hirota *et al*., 2003). Moreover, AJUBA is phosphorylated by CDK1, controls multiple cell cycle regulators, and promotes cell proliferation and tumorigenesis of colon cancer (Chen *et al*., 2016). In contrast, LIMD1 was reported to bind with the tumor suppressor Rb and repress E2F‐driven transcription to suppress cell proliferation in lung cancer (Sharp *et al*., 2004). Our study proved that WTIP is also important in regulating cell cycle and proliferation, which demonstrated the common roles of Ajuba proteins in the cell cycle and proliferation. WTIP functions much more similarly to LIMD1, a suppressive factor of cell cycle, proliferation, and tumorigenesis, than to AJUBA but acts through a different mechanism. Via GSEA, a luciferase reporter assay, western blotting, immunostaining, and so on (Fig. 6), this study systemically proved that WTIP potentiates cell proliferation and the tumorigenesis of NSCLC by attenuating AKT activity and enhancing FOXO1 expression and transcriptional activity. No interaction between WTIP and Rb has been detected (data not shown). Previous studies revealed that WTIP localized at plasma membrane, cytoplasm, and shuttled between nucleus and cytosol (Bridge *et al*., 2017; James *et al*., 2010; Rico *et al*., 2005; Srichai *et al*., 2004). Thus, accordingly, we hypothesized that there might be at least three mechanisms for WTIP inhibiting AKT signaling: (a) interacting with and inhibiting activation of some growth factor receptor(s) at the plasma membrane; (b) facilitating miRNA‐mediated gene silencing of upstream regulators of AKT; and (c) interacting with transcriptional factors or cofactors to activate or inhibit gene expression. However, how WTIP inhibits AKT is currently unclear and is an issue under further investigation in our laboratory.

## 5 Conclusion

In conclusion, this report provides mechanistic and preclinical insight into the critical role of WTIP in the regulation of the cell cycle, cell proliferation, and tumorigenesis of NSCLC through the AKT/FOXO1 pathway (Fig. S5). Our results suggest that WTIP is a tumor suppressor and may be a potential target for NSCLC treatment.

## Conflict of interest

The authors declare no conflict of interest.

## Author contributions

ZW, MQ, and ZM conceived and designed experiments and analyzed data. ZW and ZM generated the figures and wrote the manuscript. MM, YG, and XJ analyzed the data, gave comments, and revised the manuscript. JF, HW, JZ, and ZL performed experiments. DQ helped collect clinical specimens. ZY conceived the project and supervised the study.

## Supporting information


**Fig. S1.** WTIP is downregulated in NSCLC.

**Fig. S2.** WTIP inhibits cell proliferation in NSCLC cells.

**Fig. S3.** Knockdown of WTIP promotes cell proliferation in NSCLC cells.

**Fig. S4.** WTIP inhibits cell proliferation via FOXO1 signaling.

**Fig. S5.** Hypothetical model illustrating that downregulated WTIP by promoter methylation leads to activation of AKT, inhibition of FOXO1 and subsequently increased cell proliferation and tumorigenesis.

**Fig. S6.** Correlation between WTIP and WT1 signaling.
