# Supplementary figures and images for: WT1‐interacting protein inhibits cell proliferation and tumorigenicity in non‐small‐cell lung cancer via the AKT/FOXO1 axis
Source: Mol Oncol. 2019 Feb 22;13(5):1059–74. doi: 10.1002/1878-0261.12462 (PMC6487700; doi:10.1002/1878-0261.12462)

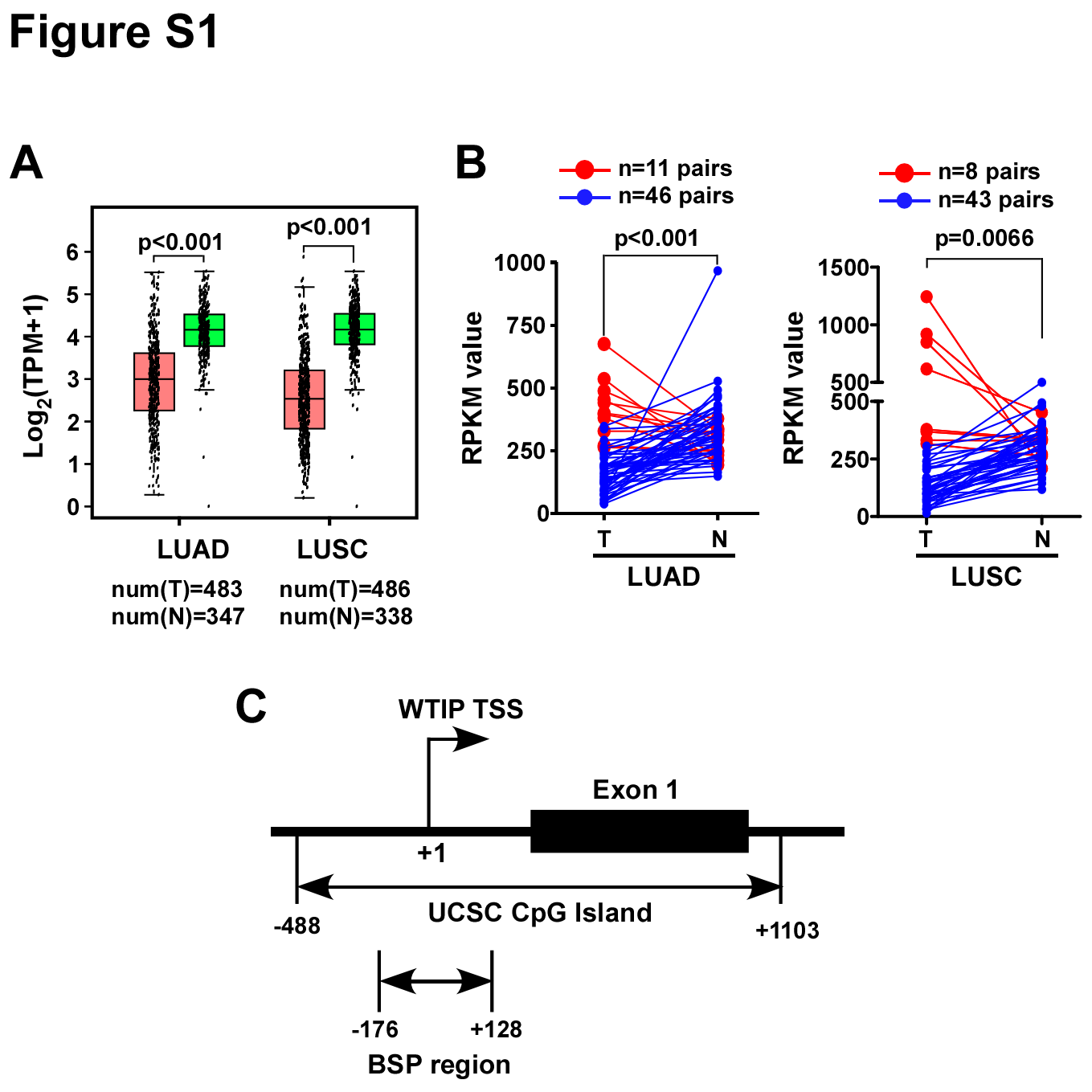

Supplement: Supplementary file 1 — Fig. S1. WTIP is downregulated in NSCLC. [file MOL2-13-1059-s001.tif]

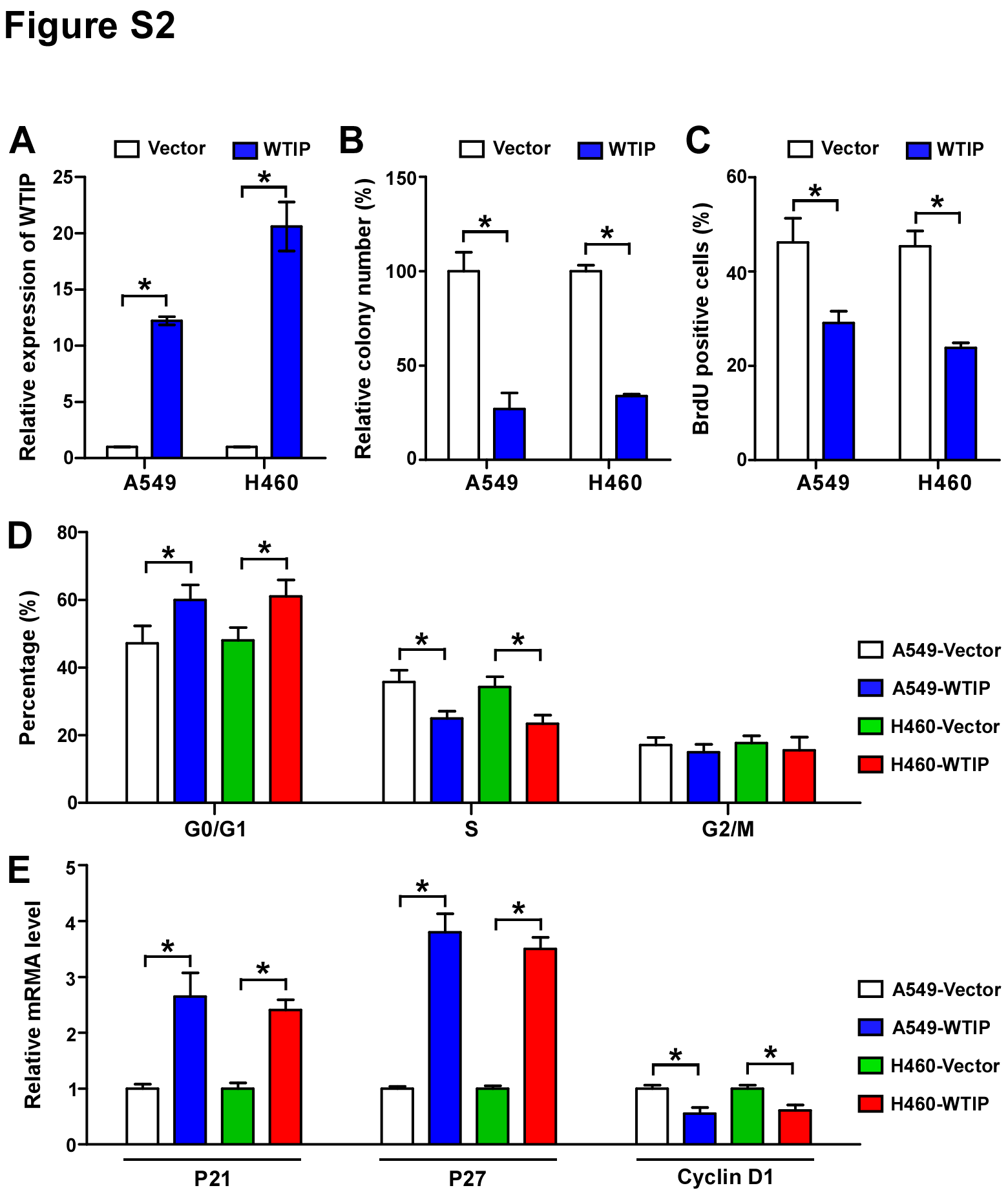

Supplement: Supplementary file 2 — Fig. S2. WTIP inhibits cell proliferation in NSCLC cells. [file MOL2-13-1059-s002.tif]

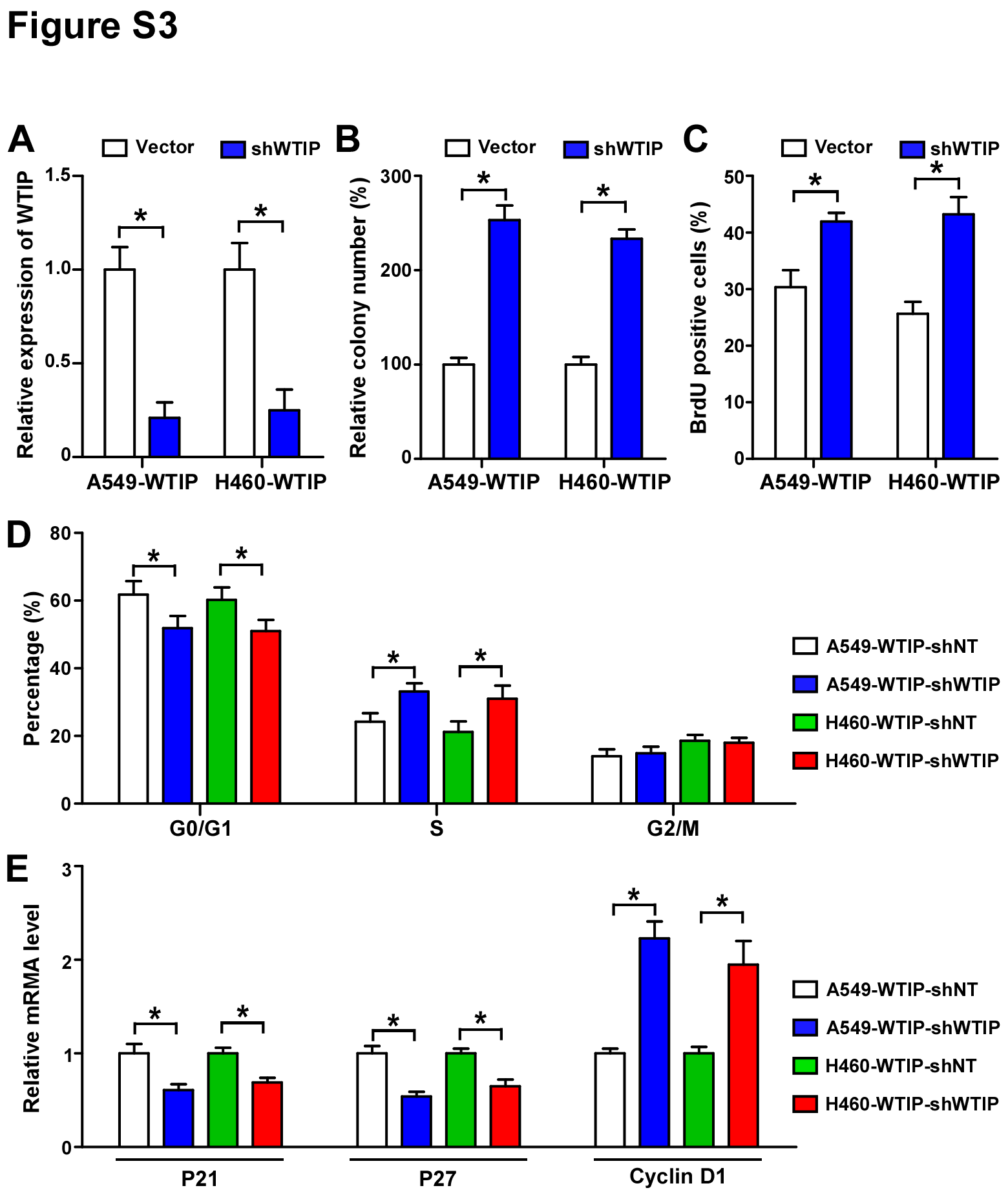

Supplement: Supplementary file 3 — Fig. S3. Knockdown of WTIP promotes cell proliferation in NSCLC cells. [file MOL2-13-1059-s003.tif]

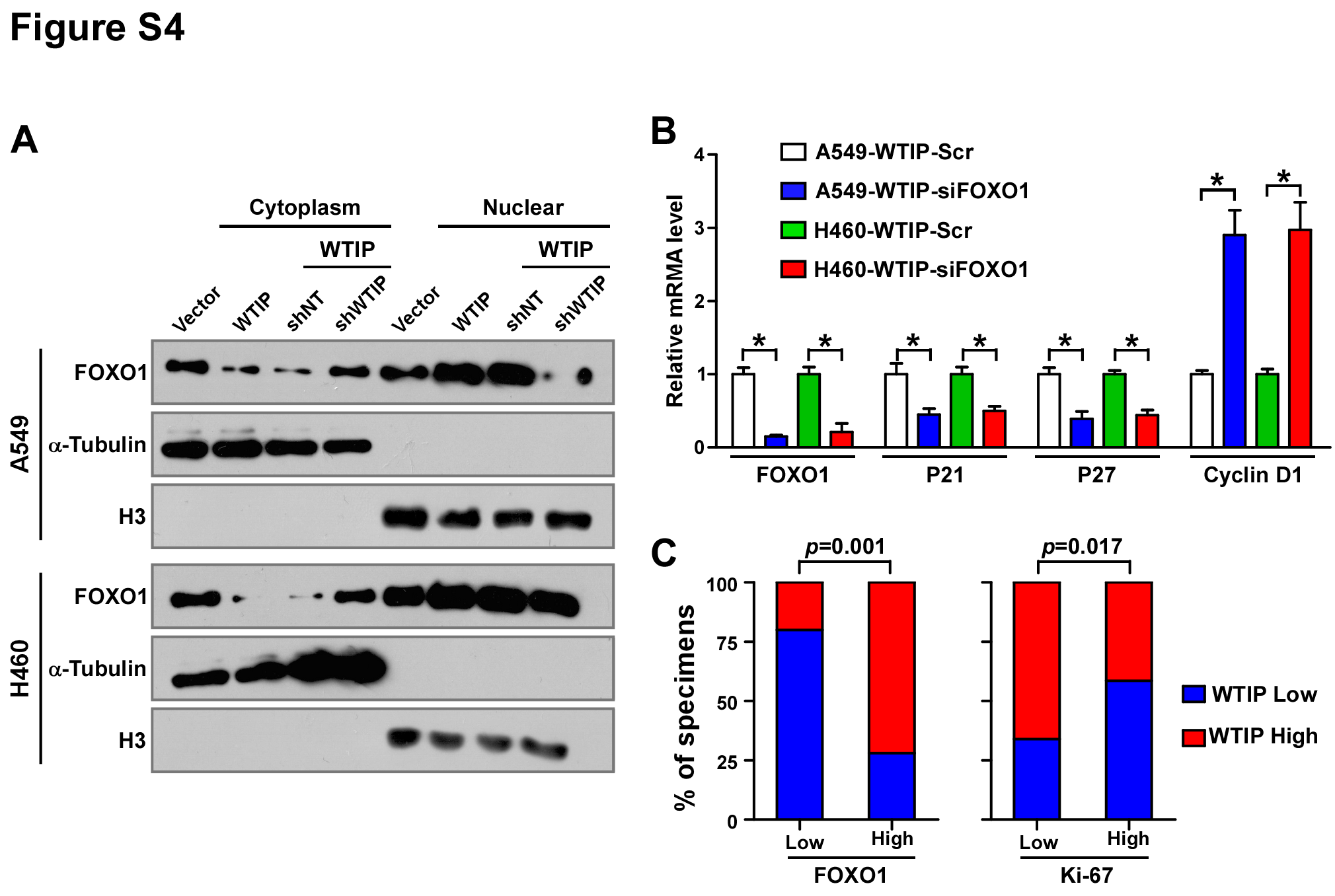

Supplement: Supplementary file 4 — Fig. S4. WTIP inhibits cell proliferation via FOXO1 signaling. [file MOL2-13-1059-s004.tif]

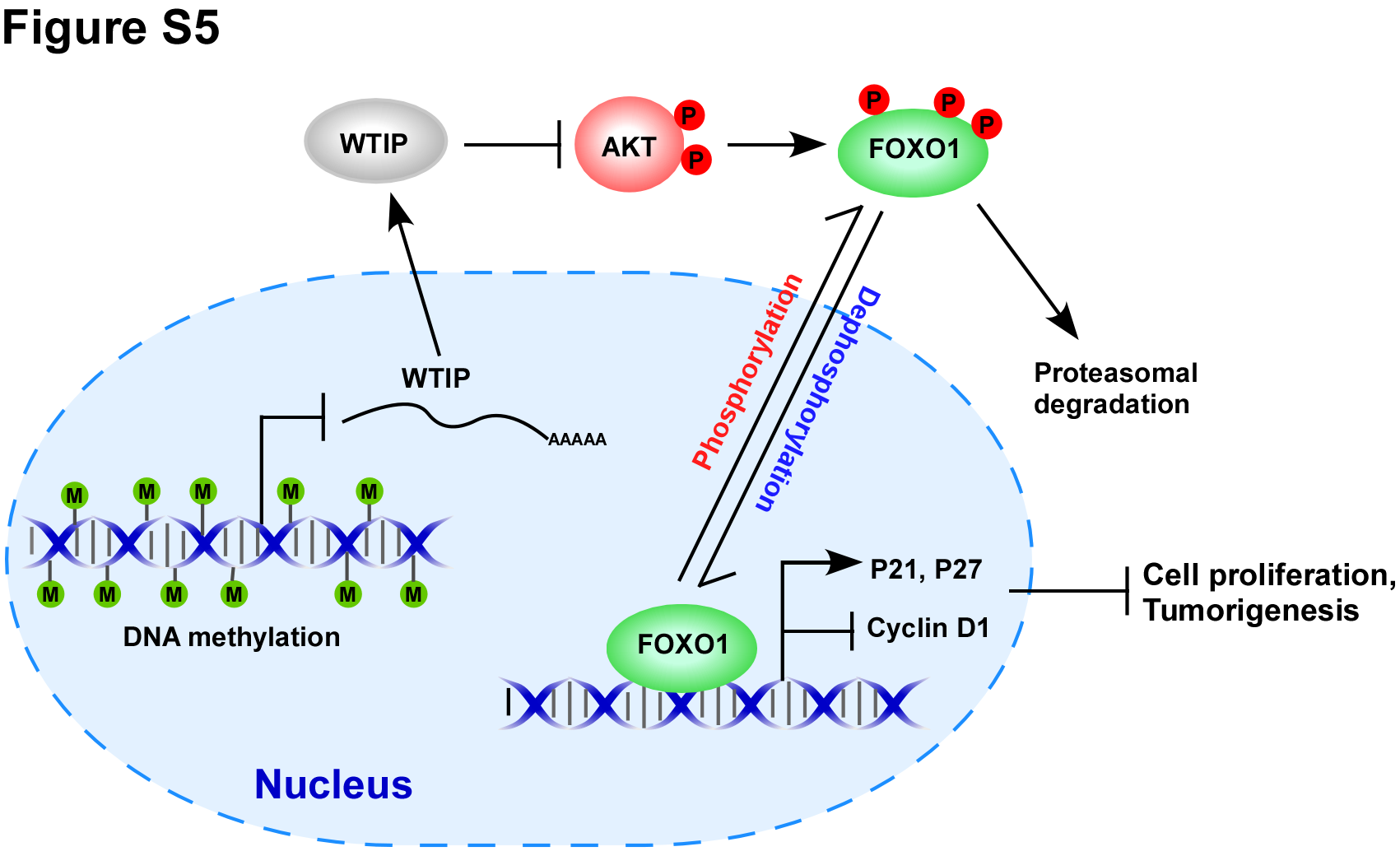

Supplement: Supplementary file 5 — Fig. S5. Hypothetical model illustrating that downregulated WTIP by promoter methylation leads to activation of AKT, inhibition of FOXO1 and subsequently increased cell proliferation and tumorigenesis. [file MOL2-13-1059-s005.tif]

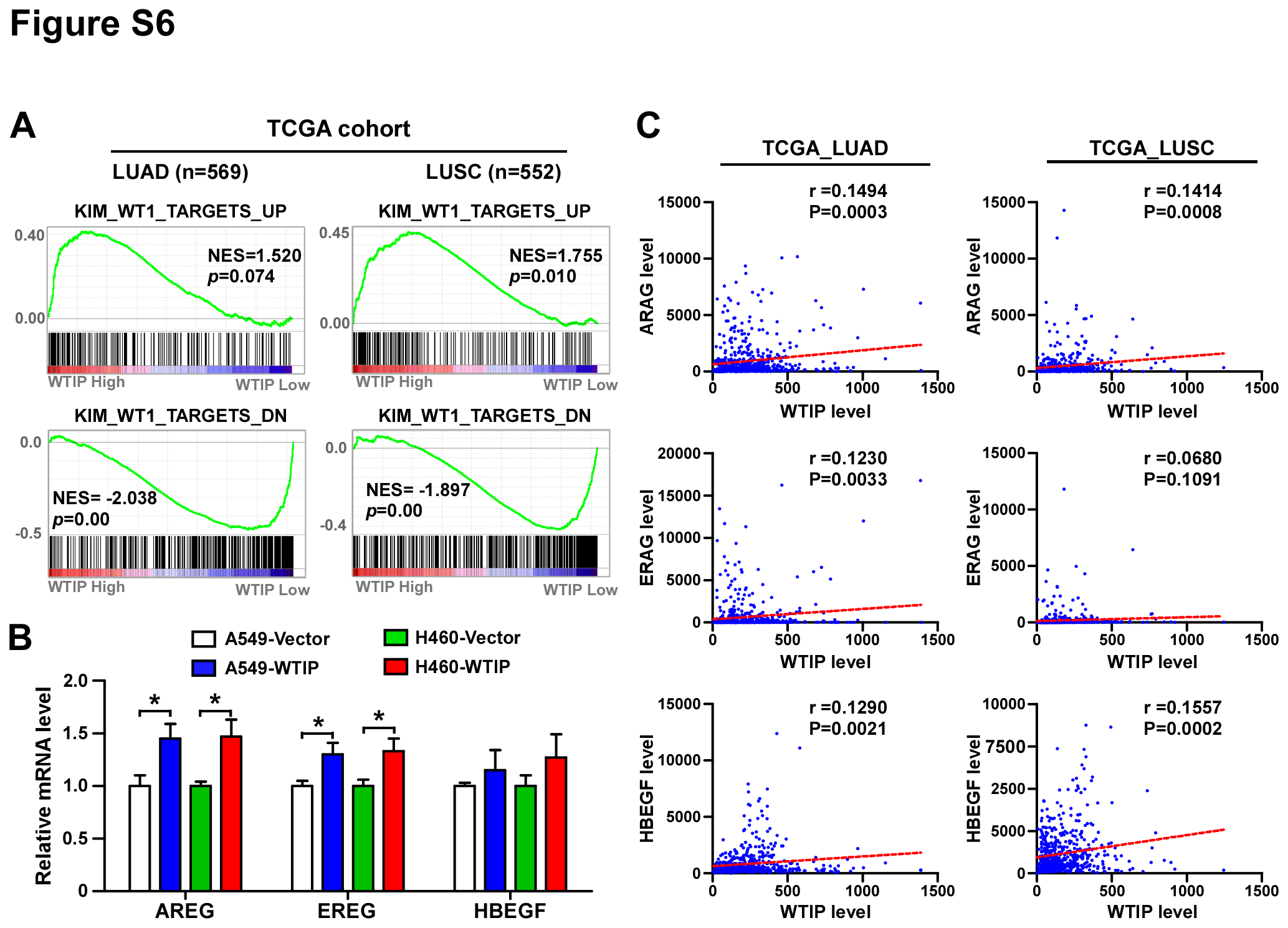

Supplement: Supplementary file 6 — Fig. S6. Correlation between WTIP and WT1 signaling. [file MOL2-13-1059-s006.tif]
