# Supplementary material for: WT1‐interacting protein inhibits cell proliferation and tumorigenicity in non‐small‐cell lung cancer via the AKT/FOXO1 axis
Source: Mol Oncol. 2019 Feb 22;13(5):1059–74. doi: 10.1002/1878-0261.12462 (PMC6487700; doi:10.1002/1878-0261.12462)
Supplement: Supplementary file 7 [file MOL2-13-1059-s007.doc]

**Supplemental figure legends**

**Figure S1. WTIP is downregulated in NSCLC.** (**A**) Expression of WTIP in LUAD and LUSC patients compared with the normal control obtained from GEPIA website (<http://gepia.cancer-pku.cn/>). Red box: tumor. Green box: normal. (**B**) Expression of WTIP in paired normal and tumor tissues of LUAD and LUSC patients of the TCGA dataset. P value was calculated by two-tailed, paired t-test. (**C**) Schematic illustration of the arrangement of the promoter region of WTIP and the region for BSP analysis.

**Figure S2. WTIP inhibits cell proliferation in NSCLC cells.** (**A**) Real-time PCR analysis of WTIP mRNA expression in indicated cells. (**B**) Quantification of cell colony formation of indicated cells. (**C**) Quantification of BrdU incorporated cells. (**D**) Quantification and statistical analysis of cell cycle distribution of indicated cells. (**E**) Real-time PCR analysis of p21, p27 and cyclin D1 mRNA level in the indicated cells. Error bars represent mean ± SD obtained from three independent experiments. *, P<0.05, unpaired t-test.

**Figure S3. Knockdown of WTIP promotes cell proliferation in NSCLC cells.** (**A**) Real-time PCR analysis of WTIP mRNA expression in indicated cells. (**B**) Quantification of cell colony formation of indicated cells. (**C**) Quantification of BrdU incorporated cells. (**D**) Quantification and statistical analysis of cell cycle distribution of indicated cells. (**E**) Real-time PCR analysis of p21, p27 and cyclin D1 mRNA level in the indicated cells. Error bars represent mean ± SD obtained from three independent experiments. *, P<0.05, unpaired t-test.

**Figure S4. WTIP inhibits cell proliferation via FOXO1 signaling.** (**A**) Western blotting analysis of expression of FOXO1 in cytoplasmic and nuclear fraction of the indicated cells. α-tubulin and histone H3 served as loading control. (**B**) Real-time PCR analysis of FOXO1, p21, p27 and cyclin D1 mRNA level in indicated cells transfected with scramble negative control or FOXO1 specific siRNA. Error bars represent mean ± SD obtained from three independent experiments. *, P<0.05, unpaired t-test. Scr, scramble. (**C**) Percentages of specimens with high and low levels of FOXO1 and Ki-67 expression in tumor tissues from patients with low or high expression of WTIP and the correlation between the expression of FOXO1 or Ki-67 and WTIP. Chi square statistical analysis was performed.

**Figure S5.** Hypothetical model illustrating that downregulated WTIP by promoter methylation leads to activation of AKT, inhibition of FOXO1 and subsequently increased cell proliferation and tumorigenesis.

**Figure S6. Correlation between WTIP and WT1 signaling.** (**A**) GSEA analyzing the correlation of WTIP expression with WT1 regulated genes in the gene expression profiles of LUAD and LUSC patients from the TCGA database. (**B**) Real-time PCR analysis of AREG, EREG and HBEGF mRNA level in the indicated cells. Error bars represent mean ± SD obtained from three independent experiments. *, P<0.05, unpaired t-test. (**C**) Linear regression analysis between WTIP and AREG, EREG and HBEGF in the gene expression profiles of LUAD and LUSC patients from the TCGA database.
